# Nursing escape rooms: Adding fun to learning during high fidelity simulations

**DOI:** 10.1002/nop2.1287

**Published:** 2022-07-13

**Authors:** Kasim Yaqub Bashir Raja, Sharon Kempson, El Pujeh

**Affiliations:** ^1^ University of Wolverhampton Wolverhampton UK

Over time, the introduction of various tools such as escape rooms, especially in medical education has come in to prominence and research in the UK (United Kingdom) and internationally is available. However, from the research completed in nursing education there is the use of escape rooms globally, but its use is finite in the UK. Studies completed internationally do not significantly focus on students' and staff perspectives or understand feelings, for example the fun element, efficacy and routes to which simulated escape rooms can be expanded to enhance students' experiences. Therefore, this study is unique, and this editorial signifies why this study is required to add knowledge to pre‐existing global research but to add knowledge from a UK perspective to enhance validity.

We believe that the introduction of multiple nursing escape rooms embedded into live and time constrained, face to face simulations would be a game changer to the UK and global nursing education, as it would offer an alternative means of educational support. The use of simulation is increasing especially with the NMC (Nursing and Midwifery Council – regulatory body for the UK nursing associates, nurses and midwives – the NMC hold the register for these professionals who are currently practising) increasing the hours of practice that can be attained via simulation. This is important as the use of simulated escape rooms could help support with adding an element of gamified fun to high intensity simulations that could potentially prove valuable as student nurses transition towards becoming a registered practitioner (Kubin, 2020). Additionally, the use of simulated escape rooms would involve providing various experiences to students that students may not have faced on placement and to support the building of confidence (Kubin, 2020).

An escape room is where individuals are in a room for a set amount of time and they have to solve a series of puzzles to gain codes to unlock the door(s) or pad lock(s). In this case, the ideology is to give students scenarios, with a time frame and codes to attain via the numbers attained through understanding the simulated case study, for example how often does a pulse check take place during in‐hospital cardiac arrest, the answer gained alongside answering further questions shall help towards a code to open the pad lock to escape the room (Kubin, 2020). The intentions are that this strategy can be used in various case studies such as cardiac arrest, anaphylaxis, and sepsis. This shall be helpful towards motivating and training students' under timed pressures that shall support learning in a safe, yet, anecdotally effective, experiential and engaging manner (Kubin, 2020).

Furthermore, promoting the use of service user engagement can be vital to nursing practices as it provides students with an “expert patient” that has clinical relevancy which shall enable greater quality interactions to ensure realism (Ferri et al., 2019). This has the potential to enhance empathy that shall also enhance patient and student satisfaction (Ferri et al., 2019). The pressures generated from the cases and time constraints may evoke feelings that are similar to the real‐life setting and studying this further shall allow us to identify whether students experiencing simulated escape rooms feel the same as in a real‐life setting. But it will also serve students with skills to become more situationally aware, prompter at giving good evidence‐based care and improve their time management (Ferri et al., 2019; Kubin 2020).

Shifting from traditional teaching of skills and simulation towards simulated escape rooms can not only promote further student centredness but also allow inclusiveness through providing students with a voice by acknowledging what students want and how we can accommodate for this, potentially, through an escape room. However, the relative lack of UK‐based research focussing on this area of expertise makes this area a very interesting and significant area of research especially when the authors have a very keen passion for this as a progressivist research project.

The concept of “Fun theory” has increased in recent years in many businesses world‐wide. Fun Theory is fundamentally a type of learning in which student nurses' behaviour can change due to the felt experience during the session (Horsley, 2010). There is evidence of the benefits of “Serious Fun” or “games,” such as higher learner participation, motivation and improved engagement, can help students link theory to practice and aid with making problem‐solving more interesting (Horsley, 2010). Rather than memorizing or learning skills for an assessment, fun theory changes behaviours in a simple, yet practical way and this can potentially help achieve better outcomes that promote lifelong learning (Kubin, 2020).

From the extrapolation of escape room research, creating a simulated escape room for students to engage and interact in has the potential to create a conducive learning environment where students' will be able to enact skills, attitudes, portray knowledge and behaviours in a high fidelity yet safe environment to make mistakes (Kubin, 2020). Learning and teaching using simulated escape rooms would therefore equip students with essential experiences and leadership that would enable them to have the confidence to become a better practitioner (Brown, Darby & Coronel, 2019). The key benefits of simulated escape rooms in nursing are that it enables students' to think critically, creatively and to be able to prioritize (Kubin, 2020). Anecdotally, it shall enhance clinical reasoning, problem‐solving, decision‐making and leadership skills through interactive engagement with colleagues by applying themselves to simulations, reflecting on outcomes (Kubin, 2020) and enhancing their knowledge through debrief and feedback.

There are potential disadvantages in terms of demands of simulated escape rooms especially in terms of resource intensity, group sizes, time consumption and giving everyone an opportunity, which could pose threats to equity (Kubin, 2020). However, it is important to consider these innovative strategies to learning as they could help towards better retention of information which shall enable longer lasting impact when compared to didactic teaching approaches.

This editorial has been written because having gained experience in simulation and completed some planning around how simulated escape rooms will work signifies its potential to bring about significant change within nursing education on a global level. Furthermore, it is crucial to put our students at the heart of the learning taking place, because the simulated escape rooms embody a multi‐faceted approach towards not just teaching nursing, anatomy, physiology, pathophysiology and pharmacology but to also help towards enhancing team‐working and communication (Brown, Darby & Coronel, 2019; Kubin, 2020). The need to understand feasibility of using escape rooms embedded amongst nursing simulation is rare but definitely possible. The potential research outcomes for nursing escape rooms can hypothetically open opportunities to implement and research upon multi‐disciplinary escape rooms. The research available around nursing escape rooms focusses on various other countries with little research in the UK, and, therefore, it is vital to implement active learning and teaching strategies that need to be researched upon to identify its efficacy. The questions that we endeavour to address as part of the research study are:

Does the embedding of nursing escape rooms amongst nursing simulations work?

What are the staff and student perspectives of implementing simulated escape rooms and should it be an integral part of the wider nursing educational strategies?

Do students feel that simulated escape rooms make learning nursing skills and theory fun? And how does the experience make them feel? Does it help towards reducing anxiety in potential real‐life settings?

Do students believe that simulated escape rooms contribute towards motivating them to enhance their knowledge during and after escape room sessions? Would students' like to have more of this? If so, why?

How can we ensure as nurses, lecturers, researchers and creators that support simulated escape rooms promote utilization of this strategy more widely across multiple hospital trusts and amongst multiple higher educational institutions to make learning and teaching fun as well as consistent?

The findings shall hopefully open more avenues to working locally, nationally and internationally with multiple institutions, to find ways in which multi‐disciplinary simulated escape rooms can be implemented in the future.

## CONFLICT OF INTEREST

There are no conflicting interests to declare.

## ETHICAL APPROVAL AND PARTICIPANT CONSENT

Ethical approval and participant consent are not required.

## Data Availability

Data sharing is not applicable to this article as no data sets were generated or analysed during the current study.
